# Pathogen Recognition by CD4 Effectors Drives Key Effector and Most Memory Cell Generation Against Respiratory Virus

**DOI:** 10.3389/fimmu.2018.00596

**Published:** 2018-03-26

**Authors:** Priyadharshini Devarajan, Michael C. Jones, Olivia Kugler-Umana, Allen M. Vong, Jingya Xia, Susan L. Swain

**Affiliations:** Department of Pathology, University of Massachusetts Medical School, Worcester, MA, United States

**Keywords:** CD4 T cells, ThCTL, T_FH_, immune memory, influenza, virus, pathogen recognition

## Abstract

Although much is known about the mechanisms by which pathogen recognition drives the initiation of T cell responses, including those to respiratory viruses, the role of pathogen recognition in fate decisions of T cells once they have become effectors remains poorly defined. Here, we review our recent studies that suggest that the generation of CD4 T cell memory is determined by recognition of virus at an effector “checkpoint.” We propose this is also true of more highly differentiated tissue-restricted effector cells, including cytotoxic “ThCTL” in the site of infection and T_FH_ in secondary lymphoid organs. We point out that ThCTL are key contributors to direct viral clearance and T_FH_ to effective Ab response, suggesting that the most protective immunity to influenza, and by analogy to other respiratory viruses, requires prolonged exposure to antigen and to infection-associated signals. We point out that many vaccines used today do not provide such prolonged signals and suggest this contributes to their limited effectiveness. We also discuss how aging impacts effective CD4 T cell responses and how new insights about the response of aged naive CD4 T cells and B cells might hold implications for effective vaccine design for both the young and aged against respiratory viruses.

## The Generation of Memory CD4 T Cell Responses to Influenza

An effective immune response to respiratory pathogens, such as Influenza A virus (IAV), requires coordination between the innate and adaptive immune system. Host defense to influenza infection begins when lung airway epithelial cells, dendritic cells (DC), and alveolar macrophages alert the host to the presence of virus through the activation of pattern recognition receptors (PRR) (1). This triggers the production of inflammatory cytokines, which activates antigen-presenting cells (APC). APC migrate to secondary lymphoid organs where they present antigen to T and B cells as soon as 2 days postinfection (dpi) (2). In a primary infection, CD4 and CD8 effectors are the most important for clearing virus, with Ab arising later. Effectors soon contract and a cohort become memory T cells that can persist in the host long-term and provide durable protection against the same virus.

Thereafter, re-encounter with the same virus goes largely unnoticed since neutralizing long-lived Ab, produced by long-lived plasma cells (LLPC), rapidly clears virus. However, when virus surface proteins hemaggluttinin (HA) and neuraminidase (NA) can mutate sufficiently and escape recognition by the Ab. IAV also escape when new strain with a distinct HA and NA subtype (heterosubtypic) develops or by a high dose of exposure that overcomes Ab. When virus is incompletely cleared, memory T cells and B cells are induced to mount a secondary response, making secondary effectors that can protect against a broader range of influenza strains (3). Most studies have focused on T cell activation during priming, but it is unclear what continued signals are needed into the effector phase to generate memory. Although live virus is cleared between 10 and 13 dpi, viral antigen presentation is detected for up to 21 dpi (4), indicating Ag recognition could continue to drive T cell responses.

Indeed antigen presentation through 8 dpi drives effector CD8 T cells to expand and promotes memory formation (5, 6). We found that antigen presentation throughout the effector response was required for almost all CD4 T cell memory generated by IAV infection (7, 8). Antigen recognition induced autocrine CD4 effector IL-2 production that acted at days 6–8 postinfection at this “checkpoint” to downregulate Bim and to upregulate the IL-7Ra. These signals along with antigen recognition, promoted survival of CD4 effectors and drove their transition to memory (7, 8). Infection was not required at this stage, since antigen-pulsed APC were sufficient to support optimal memory CD4 T cell generation and maintenance that was able to protect a naïve host from an otherwise lethal influenza challenge (7). Antigen engagement at the memory checkpoint specifically upregulated the expression of CD25, Bcl-6, and phosphorylated STAT3 in the effector CD4 T cells (8). CD25 expression is needed for efficient IL-2R signaling, so that IL-2 can prevent effector cell death (9). Costimulation through CD27 is also important at this juncture (7) for the most efficient formation of CD4 memory, just as it is during priming (10). Correspondingly, a subset of DCs that express CD70 post-priming has also been identified which correlate with CD27^+^CD8 effector expansion late into the IAV response (11).

**Figure 1:**
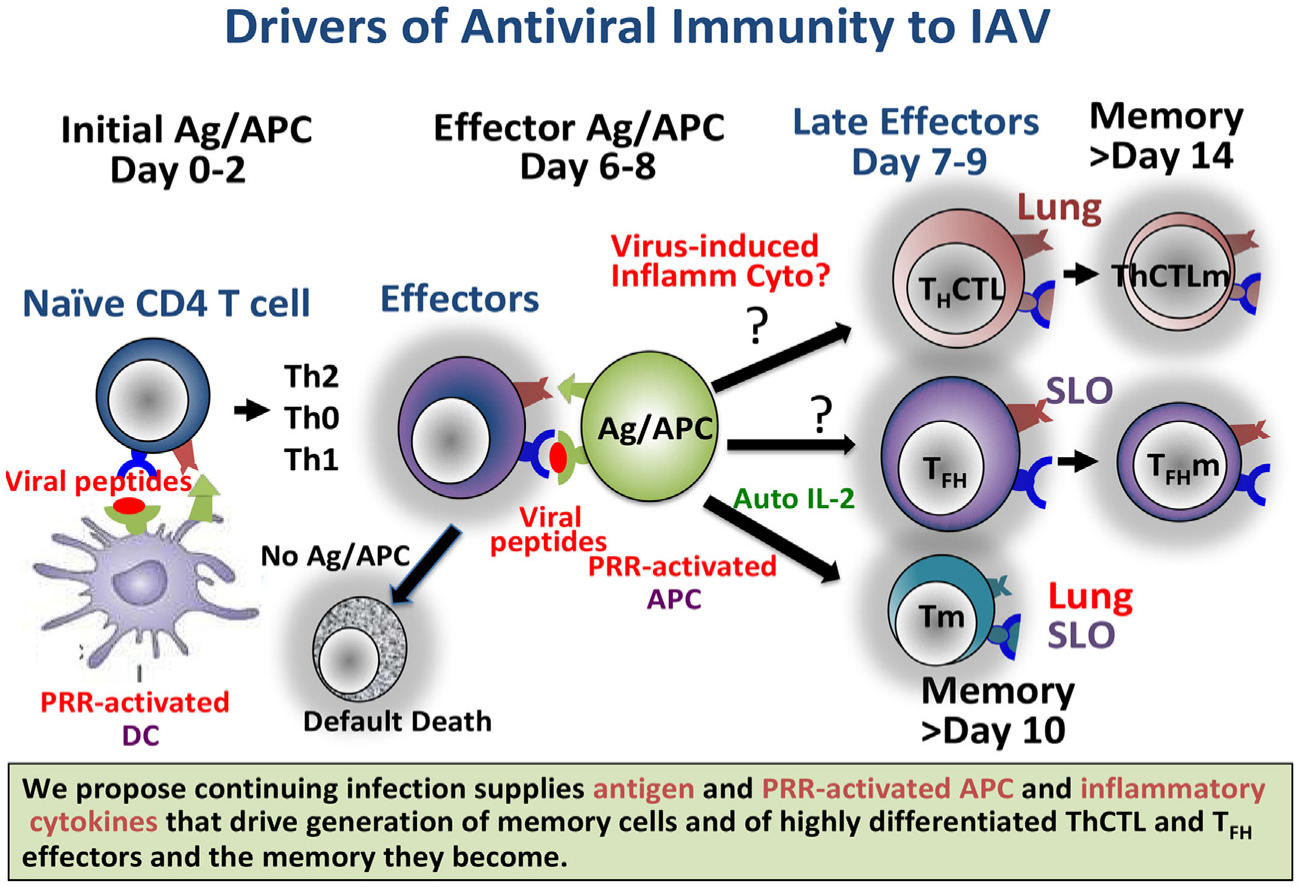
Continuing infection, producing abundant antigen and pattern recognition receptor (PRR)-activated APC, and we propose inflammatory cytokines, drives generation of memory cells and of highly differentiated ThCTL and T_FH_ effectors and the memory they become. Providing such signals in a vaccine setting should result in longer-lasting more protective immunity. Known and likely roles for viral Ag and for PRR in the response to Influenza A virus are depicted.

The need for cognate interaction of effectors with Ag–APC during this phase potentially allows for the influence by Ag dose/avidity and by co-stimulatory ligands on the APC dependent on pathogen-associated molecular patterns (PAMPs). This ensures that strong memory develops only when there is no longer infection at the early effector phase, when necessary because of unresolved threats (Figure 1). We suggest that defining pathways that drive optimum CD4 and CD8 T cell memory will inform vaccine design to produce more protective T cell memory.

## Tissue-Restricted Effector Responses Play Key Roles in Antiviral Immunity

CD4 T cells responding to viral infections differentiate into a heterogeneous population of effector cells, with subsets that mediate viral clearance through distinct mechanisms including those that are cytokine-mediated (Th1, Th2, Th17) and that kill infected cells (ThCTL), as well as indirect mechanisms of help for B cell differentiation (T_FH_) in germinal centers (GC) (3, 9, 12). Protection against lethal challenge with influenza can be mediated by synergy of CD4 T effectors acting *via* generation of Ab and by perforin-mediated lysis due to ThCTL (13).

It is well established that T_FH_ are required for GC formation and that they support GC B cell responses leading to isotype switching, somatic hypermutation, and selection of high affinity with the production of LLPC and memory B cells (14). As they recognize antigen on GC B cells, the T_FH_ in turn become GC-T_FH_ and later can become memory T_FH_ (15, 16). The LLPC are responsible for producing the long-lived Ab that provides most rapid protection against viral infection. Thus, the tissue-restricted recognition of Ag by T_FH_ is critical to GC-T_FH_ development, for subsequent T_FH_ memory and for long-lived Ab-mediated protection. The critical T_FH_ functions and their transition to memory have been well reviewed (14–16). Understanding what signals from cognate interaction of T_FH_ and GCB are needed and how long they are needed is crucial to maximizing immunity.

Another tissue-restricted CD4 effector population is the cytotoxic CD4 T cells, ThCTL (17, 18). ThCTL lyse target cells by the same mechanisms utilized by cytotoxic CD8 T cells, including the perforin-mediated pathway. ThCTL are generated during influenza and many other viral infections (19). After IAV infection they are found in the lung and bronchoalveolar lavage (19, 20), suggesting they are restricted to the sites of infection. Two markers of ThCTL have been found: CRTAM and NKG2C/E. MHC Class I-restricted T cell associated molecule (CRTAM) can be expressed by IAV-specific CD4 T cells upon activation. CRTAM^+^ CD4 cells, upregulate expression of granzymes and display peptide specific cytotoxicity, indicating these cells are ThCTL (21). While CRTAM marks cytotoxic CD4 T cells, its expression requires *ex vivo* activation, making tracking ThCTL more difficult.

NKG2A/C/E is a family of C type lectin receptors found on NK cells and CD8 T cells (22, 23). NKG2C/E, however, can be found on CD4 T cells directly *ex vivo* from infected mouse lungs (20). Isolating CD4 T cells based on their expression of NKG2C/E, indicates that cytotoxic activity *ex vivo*, as well as increased expression of perforin and granzyme is found only in the NKG2C/E-expressing effectors. NKG2C/E^pos^ ThCTL, readily degranulate and secrete high levels of IFNg when they recognize Ag, consistent with their potent antiviral activity (20). Although effector CD4 T cells infiltrate the tissues throughout the body (24), ThCTL are only found in the lung and we find that they upregulate a gene expression program consistent with tissue residency (20). Further, Ab to CD4 injected intravenously to assess relative accessibility at the effector stage, indicates most ThCTL are inaccessible, suggesting they are within the lung tissue (20). This location in sites of infection is important as local lung-resident memory CD4 T cells promote better protection than splenic memory CD4 cells against IAV infection (25).

Due to their protective capacity, in both mice and humans infected with influenza (13, 26, 27), it is important to identify the factors that support generation of ThCTL. ThCTL do not require standard polarizing cytokines during initial activation, but do depend on IL-2 (17). We find that CD4 T cells need Blimp-1 as a transcription factor to enable the cytotoxic phenotype. Loss of Blimp-1 in CD4 T cells leads to reduced ThCTL in the lungs (20), and, in others studies, reduced ability to prevent weight loss after influenza (26). Bcl-6, a transcriptional repressor of Blimp-1, is in contrast critical for T_FH_ generation (28–30), underlining the diverse transcriptional regulation of ThCTL versus T_FH_. We note that the restriction of ThCTL to sites of infection and their late appearance (20) suggest that they, like T_FH_, may require late cognate interactions to direct their final differentiation (Figure 1).

## Innate Responses Regulate the Generation of the Antiviral CD4 T Cell Response

The innate immune system plays a critical role in initiating the cascade of the adaptive responses to viruses. Toll-, RIG-I- and Nod-like receptors (TLR, RLR, or NLR) on the initial infected cells are triggered by PAMP to signal the first wave of inflammatory cytokine production. These in turn activate various APC to effectively initiate T cell priming. IAV infection activates innate pathways primarily *via* the PRR, such as TLR3, TLR7, RIG-I, and Nlrp3. Previous reviews have discussed the role of PRR-signaling in IAV infection in detail (31, 32). However, little is known beyond the role of the PRR pathways acting early in priming and initiation of T cell responses (31–33). Here, we discuss recent advances in the fields of CD4 memory, T_FH_ and ThCTL that are making it clear that PRR pathways play a more global role in shaping CD4 effector and memory responses.

We find that the generation of CD4 memory does not require infection during the effector phase, as activated APC presenting peptides are sufficient to drive *in vivo* generated CD4 effectors to become memory in uninfected mice (8). However, the role of PRR pathways in generating specialized CD4 memory responses such as T_FH_ memory, ThCTL memory and CD4 T_RM_ is only now being studied. The gamma-chain cytokines, IL-2, IL-7, and IL-15, each play important roles in T cell memory (7, 34, 35). PRR pathways can induce high levels of IL-15 during infection (35). While we know that constitutive levels of IL-15 and IL-7 maintain homeostatic memory CD8 and CD4 T cell populations (35), the role of high levels of PRR-signaling such as that leading to type I IFN and other proinflammatory cytokines during active infection remains unclear. We find that IL-15 is required during the effector phase of the CD4 response for the generation of an IL-2 independent CD4 T_RM_ population (36). Another study indicates local inflammatory cues from IL-12 and IFNβ, made by intestinal macrophages, are involved in differentiation and persistence of the CD8 T_RM_ populations (37). While multiple PRR pathways promote T cell memory, the causal relationship between the memory subsets and the specific PRR has only been shown indirectly through the requirement for innate cytokines.

The TLR9 adjuvant CpG acts on TLR9/MyD88 signaling in both DC and B cells to promote optimum T_FH_ generation (37). Type I IFN produced by PRR-signaling, also promotes the T_FH_ genetic program. Type I IFNα/β induced Bcl-6, CXCR5, and PD-1 expression in CD4 T cells that were activated *in vitro* by Ab to CD3 and CD28, by a STAT-1-dependent pathway (38). During persistent LCMV infection, chronic Type I IFN supports T_FH_ formation (39). On the other hand, another study showed that the absence of STAT3 in CD4 T cells during an acute LCMV infection resulted in reduced T_FH_ differentiation caused by increased Type I IFN production, thus suggesting that Type I IFN indirectly inhibited T_FH_ differentiation (40). Type I IFNs have widely variable effects on T cell activation depending on whether they are present before activation, during activation or after activation and depending on whether they are present acutely or chronically (41). Thus, it is likely that the disparate impacts of Type I IFN on T_FH_ generation in the studies above are due to differences in model systems used.

CD4 T cells stimulated *in vitro* in the presence of IFNα and IL-2 have increased cytotoxic potential (26). CD4 T cells in the lungs of IFNAR-deficient mice also expressed lower levels of Granzyme B and perforin, suggesting ThCTL generation also may depend on inflammatory cytokines. In support of roles for inflammation acting on T effectors, gene profiling following IAV infection indicate innate cytokines including Type I and Type III IFNs, are produced well into the effector phase of the response (42). Since innate inflammatory cytokines, such as Type I IFN, can be pathological (43) it makes sense that their production requires PRR activation, present only during continuing infection and that they could, then help further drive the differentiation of specialized effector CD4 T cells to ensure pathogen clearance.

Innate pathways also play an indirect role in promoting continued antigen presentation by inducing inflammation and enhancing APC activation. A recent study showed that type 1 IFN has differential effects on APC subsets by inducing different levels of co-stimulatory ligands, such as various TNFSF ligands, CD80, and CD86 on inflammatory APCs versus classical DCs, which control priming of CD4 T cells (44). Expression of many of these TNFR ligands such as OX-40, CD27, and 4-1BBL have been correlated with protective antiviral T cell responses (45, 46).

## Pathogen Recognition Promotes T Cell and B Cell Immunity

In addition to the roles of PRR-stimulation in driving the final differentiation of T_FH_, discussed above, we recently showed that PRR-activated DC, acting as APC for CD4 T cells, could greatly augment both T_FH_ generation, generation of IL-21 secreting CD4 effectors. This indirectly enhances generation of GCB cells and most importantly of long-lived Ab in response to inactivated IAV vaccine (47). Vaccines, especially traditional inactivated or subunit flu vaccines often result in weak, barely protective Ab levels and low frequency T cell memory, especially when given to the elderly (48). In contrast, live infection is able to generate very long-lived and effective immunity of all types, suggesting that vaccines will need to be modified to provide the key signals inherent in live infection in order to improve efficacy. We found that when peptide-pulsed DC were activated by TLR-signaling and used to prime CD4 T cells specific for IAV, high levels of inflammatory cytokine were produced during the CD4:Ag/APC interaction. This enhanced otherwise weak helper T and B cell responses by an IL-6 dependent mechanism. This effect was apparent even in an unmanipulated mouse, where TLR-activated DC, presenting inactivated IAV, enhanced B cell IgG Ab response over several months (47). PRR activation in conjunction with BcR-triggering also can directly activate B cells and promote their differentiation to AbSC. This synergistic signaling drives T-independent B cell responses, but can also be involved in conventional B cell (B2) responses (48, 49). Thus, the presence of PRR signals acts at multiple levels in DC and in B cells to promote the initial B cell response, and we propose also later to promote T_FH_, LLPC, and memory B cells.

## Aged T and B Cell Responses Become More Dependent on PRR Pathways

Animals undergo a dramatic shift in their immune responses as they age. The number of naive T cells (50) and naive follicular B cells (FOB) decreases and remaining naive cells are less responsive (51). So while previously established T and B memory cells often remain largely functional, responses to new viruses or strains of viruses not previously encountered is compromised. Our studies show that aged CD4 T cells have reduced responsiveness to IL-6 (47, 52) leading to weak generation of helper subsets. Providing high Ag doses on PRR-activated APC, markedly enhanced aged naive CD4 helper responses and indirectly improved B cell responses by a mechanism dependent on APC produced IL-6 (47). This study provided clear evidence that PRR signals acting on APC were responsible for improved CD4 helper responses and supported the concept that greater PAMP signaling is required to prime naive T cells as they age.

We wondered if the reduced responses by B cells in the aged could also be enhanced by high Ag dose and PAMP stimulation. We also noted that T-independent B cell responses, do not require the age-compromised T helper cells, might provide immune protection in the aged. A subset of B cells termed “age associated B cells” (ABC) that lack both CD23 and CD21 increase with age in mice (53). Studies showed that some of the ABC can develop from antigen-experienced FOB suggesting they are memory-like B cells (54). However, we find that most ABC in unimmunized mice express only surface IgD and IgM and lack the expression of key B cell co-stimulatory and activation markers, suggesting they are naive (51). These sIgD^+^ ABC transferred to RAG-deficient mice, were driven by IAV infection into AbSC specific for IAV (51), indicating a T-independent pathway to Ab production. ABC identified in models of autoimmunity are dependent on BcR, TLR-7, and TLR-9 stimulation (53, 55), and memory ABC populations are implicated in mediating autoimmunity (56, 57). Thus like CD4 T cells, the ABC may require strong PRR-signaling to respond effectively. We propose that pathogens such as respiratory viruses can induce sIgD^+^ ABC to generate protective Ab responses even in the aged (51). We are investigating whether the sIgD + ABC response to IAV infection can indeed contribute to a protective Ab response and if this is dependent on high levels of Ag and PAMP signals.

## Speculations and Implications for Vaccines

It has become increasingly clear that vaccines, especially inactivated, subunit, or recombinant protein vaccines, often result in weak, barely protective Ab levels and low frequency T cell memory, and they are even less effective in the elderly (48, 58). On the other hand, LAIV are able to elicit superior lung specific responses and T_RM_ (59). Various studies have indeed shown that that local antigen presentation and local inflammation are key factors that drive tissue-resident memory (60, 61). Live infection is able to generate effective immunity of all types that often lasts several decades or more, suggesting that modifications to vaccines to provide key signals inherent in live infection should be able to improve vaccine efficacy (62). Here, we suggest that strong signals like those from replicating pathogens, including high doses of antigen persisting to the peak of the immune response, along with high levels of PAMPs acting on innate and B cells, are necessary to optimally trigger T cell effector and memory generation and B cell response. Replicating viruses that facilitate high and continued levels of antigen presentation and inflammation, in addition to tissue localization of the immune response induced by intranasal administration, may be key factors that determine superior vaccine induced protection. Though further definition of these pathways is needed to best inform vaccine strategies, we propose that they can ultimately be used to drive generation of increased immunity against respiratory viruses.

## Author Contributions

SS, PD, MJ, AV, OK-U, and JX wrote various sections in the manuscript. SS and PD conceptualized and organized the article and coordinated author contributions. MJ finalized the manuscript for submission. All other authors did experimental work that influenced the thinking behind the article. All authors contributed to manuscript revision and have read and approved the submitted version.

## Conflict of Interest Statement

The authors declare that the research was conducted in the absence of any commercial or financial relationships that could be construed as a potential conflict of interest.
